# Singlet Oxygen and Free Radical Reactions of Retinoids and Carotenoids—A Review

**DOI:** 10.3390/antiox7010005

**Published:** 2018-01-01

**Authors:** Ruth Edge, T. George Truscott

**Affiliations:** 1 Dalton Cumbrian Facility, The University of Manchester, Westlakes Science and Technology Park, Moor Row, Cumbria CA24 3HA, UK; ruth.edge@manchester.ac.uk; 2 School of Chemical and Physical Sciences, Lennard-Jones Building, Keele University, Staffordshire ST5 5BG, UK

**Keywords:** carotenoids, xanthophylls, retinoids, lycopene, pro-/anti-oxidants, singlet oxygen, neutral free radicals, radical cations/anions, hydroxyl radical, hydrogen abstraction

## Abstract

We report on studies of reactions of singlet oxygen with carotenoids and retinoids and a range of free radical studies on carotenoids and retinoids with emphasis on recent work, dietary carotenoids and the role of oxygen in biological processes. Many previous reviews are cited and updated together with new data not previously reviewed. The review does not deal with computational studies but the emphasis is on laboratory-based results. We contrast the ease of study of both singlet oxygen and polyene radical cations compared to neutral radicals. Of particular interest is the switch from anti- to pro-oxidant behavior of a carotenoid with change of oxygen concentration: results for lycopene in a cellular model system show total protection of the human cells studied at zero oxygen concentration, but zero protection at 100% oxygen concentration.

## 1. Introduction

The C_20_ retinoids and C_40_ carotenoids play important roles in many diverse biological processes. The retinoids are major pigments associated with the eye and other aspects of human health (e.g., the skin) while the C_40_ carotenoids are not only involved in vision but also in photosynthesis and play a major role as anti-oxidants in human health [1]. In this review, we discuss the anti- and pro-oxidative reactions of the C_20_ retinoids. More extensively, we compare the anti-/pro-oxidant properties of the C_40_ hydrocarbon carotenoids (subsequently called “carotenoids”) with those of the C_40_ oxygen-containing carotenoids (subsequently called xanthophylls). In biological systems there is some degree of “selectivity” between the carotenoids and the xanthophylls—thus, in the macula of the eye, there are no carotenoids but three xanthophylls (lutein, zeaxanthin and meso-zeaxanthin) [2] while in photosynthetic systems it is the hydrocarbon carotenoid, β-carotene which is the major carotenoid, although xanthophylls are also involved as accessory pigments [3]. Also, the treatment of the extreme photosensitivity associated with the disease of erythropoietic protoporphyria currently uses only β-carotene to ameliorate the damage due to singlet oxygen [4]—however, some xanthophylls have been considered (but more or less discounted) as skin colorants (artificial “tans”) [5]. The roles of all three: the retinoids, the carotenoids and the xanthophylls, as both anti- and pro-oxidants, with respect to singlet oxygen and radical generation and quenching, are discussed.

## 2. The Retinoids

**Figure 1:**
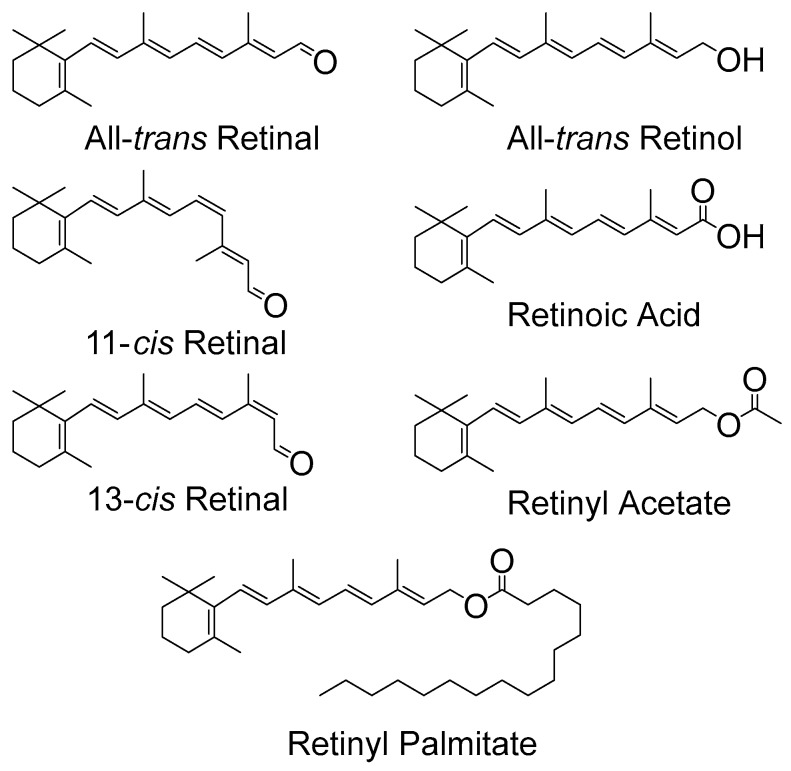
Chemical structures of several retinoids.

Retinoids have many roles in biological systems that do not involve anti- or pro-oxidant properties, such as roles in general growth, differentiation of epithelial tissues and in reproductive health. Nevertheless, there is some interest in such anti-/pro-oxidant activity, for example due to photosensitization, which is believed to involve singlet oxygen and free radical production. Topical retinoids are used to treat a wide range of dermatological disorders, however, advice to patients is that such drugs should only be used at nighttime to avoid the possibility of photosensitivity arising from activated oxygen species. The structures of several retinoids are shown below in Figure 1. 

### 2.1. Triplet States and Singlet Oxygen (^1^O_2_)

All-*trans* retinal and other isomeric forms of retinal (see Figure 1) contain five conjugated carbon-carbon double bonds and a carbonyl group. The non-bonding electrons (n) on the oxygen lead to n-π* excited states as well as the π-π* excited states from the conjugated carbon–carbon double bonds. All-*trans* retinal is, of course, the key chromophore in vision, and laser flash photolysis has been used extensively to investigate the production of its triplet states and ^1^O_2_. In addition, a simple, new method for estimating ^1^O_2_ reactivity with retinoids and carotenoids, based on DBPF (1,3-Diphenylisobenzofuran)/UV-Vis (Ultra Violet-Visible) absorption spectroscopy in micellar solutions, has recently been proposed [6]. Pulsed laser studies show a significant production of triplet states and ^1^O_2_ from all isomers of the retinals studied in non-polar solvents, including the all-*trans* isomer. Typical values for triplet and ^1^O_2_ quantum yields being around 0.2–0.5, depending on the solvent, isomeric form and even laser excitation wavelength [7,8]. Note that a quantum yield value of 1.0 is equivalent to 100% yield. The solvent dependency (triplet yields being much lower in polar solvents than in non-polar solvents) allowed the ordering of the ^1^n-π*, ^1^π-π*, ^3^n-π*, and ^3^π-π* excited states to be determined. For the retinal isomers studied in polar, H-bonding or non-bonding solvents a state of ^1^π-π* character, located below ^3^n-π*, appears to be the lowest excited singlet state. However, a ^1^n-π* state located above the ^3^π-π*, is the lowest excited singlet state in non-polar, non H-bonding solvents, so that significant ^1^O_2_ can be generated leading to photo-oxidative damage in, for example, CCl_4_ and hydrocarbon solvents. In more polar environments the ^1^O_2_ quantum yield is much lower [9], for example, in methanol values near 0.08 for all-*trans* retinal were reported via ^1^O_2_ luminescence (these authors obtained rather similar results via steady-state techniques, which may be less accurate under some conditions). However, none of the other retinoids studied (see Figure 1) have such a high triplet quantum yield in any solvent. For the visual pigment models (retinal Schiff bases) there are claims of triplets being formed but with quantum yields in the region 0.01. Furthermore, Becker and co-workers suggest, at least in some solvents, that even this small yield of triplet state formation is due to minor hydrolysis of the Schiff base to produce a small amount of retinal [10]. 

Other retinoids, without a carbonyl group (see Figure 1) and hence no n-π* states, have been studied and include retinol, retinyl acetate and retinoic acid. Triplet yields in the region 0.03 to near zero have been reported and reviewed previously, see, for example, [1]. Retinoic acid is of particular interest because of its use to treat leukemia and other cancers. However, such treatments lead to significant skin photosensitivity in at least some patients [11]. Laser flash photolysis studies have measured a triplet yield in hexane of 0.0013 for retinoic acid and this triplet was seen to be quenched by oxygen (presumably to generate ^1^O_2_) very efficiently, k = 1.4 × 10^9^ dm^3^ mol^−1^ s^−1^. Thus, despite the rather low triplet yield, there is still evidence of singlet oxygen formation. It seems reasonable to suggest the skin photosensitivity, at least to some extent, is related to ^1^O_2_ and a trial to mitigate this, e.g., with β-carotene seems worthwhile.

Retinyl acetate and palmitate are frequently used to treat disease and are also components of cosmetics, and similar photophysical properties have been reported for retinyl acetate as for retinoic acid—typically, a triplet quantum yield of 0.025 and reaction rate constant with oxygen of 1 × 10^9^ dm^3^ mol^−1^ s^−1^ [12]. Once again, there is a low triplet yield but evidence of singlet oxygen formation.

### 2.2. Retinoid Radicals

As well as via triplets and ^1^O_2_, retinoid-induced photosensitivity can also arise via radical formation and subsequent secondary processes [13]. Radical-based processes have been studied via laser flash photolysis and pulse radiolysis, however, the chemistry is complex and still not fully understood.

Certainly, while direct excitation of retinoic acid in methanol produces only the triplet (and presumably ^1^O_2_, see above) for retinyl acetate, a species absorbing near 590 nm, other than the triplet, was also observed. Rosenfeld et al. [14] proposed this species as a carbenium ion. Lo et al. [12] reported the same transient, and in agreement with Rosenfeld’s assignment, could see no solvated electron and no reaction with β-carotene (which would have been observed if photo-ionization was occurring)—the major process was proposed to be elimination of OCOCH_3_^−^

RCH_2_OCOCH_3_ → RCH_2_^+^ + OCOCH_3_^−^

to produce the retinylic carbenium ion. When water mixed with methanol was used as the solvent, some degree of photo-ionization was also observed, suggesting a balance between photo-dissociation and photo-ionization of retinyl acetate depending on the micro environment. If the solvated electron is produced, a further complexity arises with the radical anion also being formed. Pulse radiolysis has suggested the radical anion itself will then dissociate by eliminating OH^−^.

### 2.3. Radical Reactions

The superoxide radical anion (O_2_^•−^) is generally regarded as unreactive while its conjugate acid, HO_2_^•^, is much more reactive (the pK_a_ of O_2_^•−^/ HO_2_^•^ is 4.7) and it is important not to claim reactivity for O_2_^•−^ even at pH values near neutrality because this may simply be due to the small amounts of HO_2_^•^ present at such pH values. Nevertheless, Collins et al. [15] used a cyclic voltammetry/computer simulation to obtain estimates of the rate constants for the reaction of all*-trans* retinol with O_2_^•−^ and HO_2_^•^ as ≈ 4 × 10^5^ and ≥1.5 × 10^8^ dm^3^ mol^−1^ s^−1^. 

Rozanowska and colleagues [16] have reported pulse radiolysis studies of the interaction of retinal, retinol and retinoic acid with peroxyl radicals. The rationale for this work is that the assumed key process for their ability to inhibit lipid peroxidation, via the formation of carbon-centered radical adducts, is not sufficient to fully explain the high effectiveness of retinol and retinoic acid to inhibit lipid peroxidation, which can even exceed that of vitamin E. In order to further understand such protection, pulse radiolysis was used to generate peroxyl radicals and the reactions between the retinoids and peroxyl radicals were monitored in aqueous micelles. In all cases, at least two products were detected—the retinoid radical cation (absorbing near 590 nm) and species absorbing at shorter wavelengths, which are probably adducts of the retinoids with the peroxyl radicals. The subsequent processes of these “adducts” suggests that the mode of interaction of different retinoids with peroxyl radicals may vary.

Rozanowska et al. suggest the donation of an electron from the retinoid to the peroxyl radical:

CCl_3_O_2_^•^ + Retinoid + H^+^ → Retinoid^•+^ + CCl_3_O_2_H

this provides an additional route for the anti-oxidant action of retinoids, provided that the formed radical cation is itself subsequently removed by a reducing species such as vitamin C—such a process for the carotenoids was observed [17]. 

El-Agamey and co-workers [18] reported kinetic studies of retinol addition radicals formed with various thiyl radicals, with and without oxygen, in an attempt to quantify the pro-oxidative effects of retinol. The reactions observed in methanol are neutral thiyl radical (RS^•^) additions to retinol and the estimated rate constants are the sum of the individual addition rate constants leading to the formation of various thiyl addition radicals. Typically, these were 2 − 8 × 10^9^ dm^3^ mol^−1^ s^−1^, rather similar to that previously reported for β-carotene [19]. Oxygen addition reactions are important in understanding the anti-/pro-oxidation balance of retinoids and, if oxygen addition to the adducts is significant (to give R–CAR–OO^•^), then retinoids may well switch from being anti-oxidants to pro-oxidants. El Agamey et al. found that such oxygen addition processes for the thiyl-retinal neutral adducts are 10–1000 times larger (depending on the reactivity of the specific thiyl radical) than those reported for the carotenoids [20,21]. From these results, under the conditions studied by these workers, carotenoids may be the more potent anti-oxidants and, correspondingly the retinoids more potent pro-oxidants.

El-Agamey and Fukuzumi [22] used laser flash photolysis to study retinol in polar solvents (mainly methanol) and showed that 355 nm excitation leads to the formation of the retinol radical cation (λ_max_ = 580 nm) and the solvated electron. The electron then adding to the parent retinol to generate the corresponding radical anion (λ_max_ = 370 nm). In particular, the 580 nm species was proven to be the radical cation (and not the corresponding non-radical retinyl cation RCH_2_^+^). Using this identification of the absorption maximum for the retinol radical cation, El-Agamey and co-workers have studied the reactivity of this radical cation with a very wide range of organic and biological molecules [23]. The systems studied included C_40_ carotenoids, vitamins C and E, amino acids and natural and synthetic phenols, neurotransmitters such as catechols and various phenols. Their results, comparing rate constants with those of CCl_3_O_2_^•^, showed that the reactivity of the retinol radical cation is greater or similar to that of CCl_3_O_2_^•^, i.e., retinol radical cation is an extremely powerful oxidizing species and can be expected to cause bio-damage. However, their results also showed that the presence of vitamins E and C, C_40_ carotenoids, and naturally occurring phenols (e.g., L-dopa, vanillin and reservatrol) can inhibit the potentially damaging effects of the retinol radical cation by reducing it to retinol.

## 3. Carotenoids

**Figure 2:**
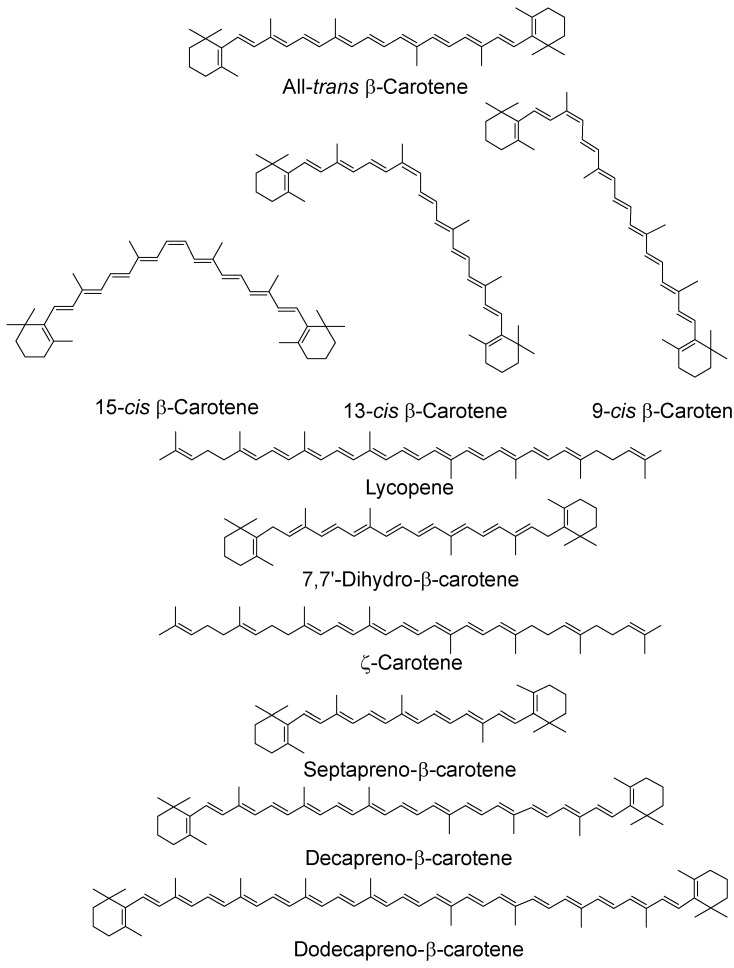
Chemical structures of typical carotenoids; *cis* isomers have been given for β-carotene only.

**Figure 3:**
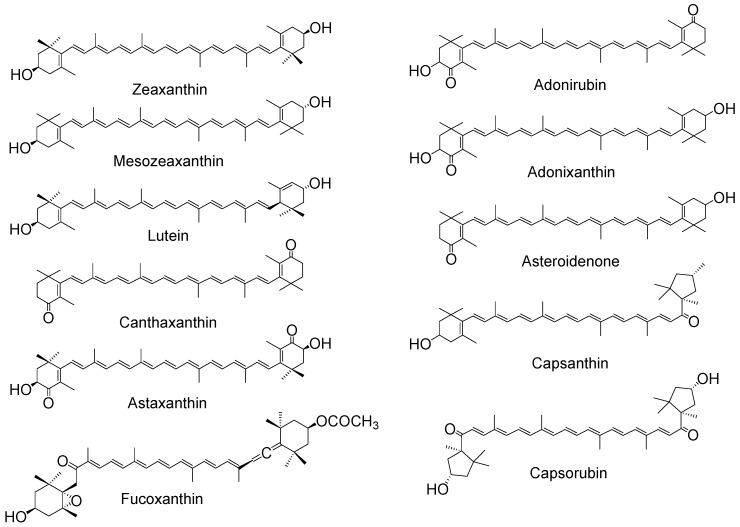
Chemical structures of typical xanthophylls.

The anti-/pro-oxidant roles of carotenoids and xanthophylls (representative structures shown in Figure 2 and Figure 3, below) are of particular interest to photosynthesis and vision. In photosynthesis their roles include protection of the reaction centers and the antenna complex. In vision the xanthophylls protect the macula from light-induced damage via a simple blue light filtering mechanism and, probably, also via quenching of reactive oxygen species, such as free radicals and singlet oxygen. Much interest has centered on the beneficial and possible deleterious effects of using both carotenoids and xanthophylls as dietary supplements against diseases such as cancer and age-related macular degeneration, which is the major cause of blindness in older people in the western world.

Carotenoids are one of the most common pigments found in nature, being responsible for the red/yellow colors of many leaves, fruits and fish. Epidemiological work has associated their dietary intake with a reduced risk of degenerative diseases but supplement trials have suggested pro-oxidant abilities.

### 3.1. Carotenoids and Singlet Oxygen

Ground state molecular oxygen is a triplet state with the two unpaired electrons being in the degenerate pair of π* orbitals. The two lowest electronic excited states of oxygen in the gas phase are both singlet states and it is the lowest lying that is commonly termed singlet oxygen (^1^O_2_) [24].

Singlet oxygen can be produced in a number of ways, e.g., peroxide decomposition, high frequency discharge and via energy transfer from the excited state of a photosensitizer to ground state molecular oxygen [24], which is the most common method. The relatively low energy level of ^1^O_2_ (E = 0.98 eV or 94.5 kJ mol^−1^) means that a wide range of sensitizers have a high enough energy in both their singlet and triplet states to convert molecular oxygen to its excited state, ^1^O_2_. Typical in vivo sensitizers are porphyrins, chlorophylls and riboflavin, which can lead to a variety of deleterious effects, including DNA damage and lipid peroxidation [25,26]. Other sensitizers include dyes such as rose bengal and eosin, which are often used in organic solvents or in ex vivo cell suspensions to produce ^1^O_2_.

Once produced ^1^O_2_ is capable of oxidizing many cellular substrates, especially in the skin and eyes, where photo-production can occur [27,28], but it has a limited lifetime and, if no reaction occurs, will decay back to the ground state either radiatively or by solvent-induced non-radiative deactivation. The non-radiative process dominates in solution and so the ^1^O_2_ lifetime is strongly influenced by the solvent vibrational frequencies, varying from a few microseconds to several milliseconds (compared with a half-life of 45 min in the gas phase [29]). The radiative component of the deactivation of ^1^O_2_ (phosphorescence) has a maximum around 1270 nm and this decay is often used for monitoring ^1^O_2_ and determining quenching rate constants of antioxidants.

In fluid systems/solvents carotenoids quench ^1^O_2_ physically via collisional energy transfer, and there have been studies of both their quenching abilities and of their protection against ^1^O_2_-mediated photo-oxidation reactions. 

Foote and Denny [30] were the first to show inhibition of photosensitized oxidation by β-carotene and that it was due to its ability to efficiently quench ^1^O_2_. Farmilo and Wilkinson [31] showed that (electron exchange) energy transfer quenching is the principal mechanism of carotenoid ^1^O_2_ quenching, producing the carotenoid triplet state. Chemical quenching (which destroys the carotenoid) only occurs as a much slower process leading to mainly carotene endoperoxides [32,33,34].

As noted above, many carotenoids have been studied with regard to their ^1^O_2_ quenching ability in organic solvents and it has been shown that ability increases with increasing number of conjugated double bonds and, therefore, increasing wavelength of the ππ* absorption maximum [35,36]. For the biologically important C_40_ carotenoids the triplet energy is below that of ^1^O_2_ and all quench at near-diffusion-controlled rates of around 1 × 10^10^ dm^3^ mol^−1^ s^−1^. Longer chain carotenoids, such as decapreno-β-carotene and dodecapreno-β-carotene, have quenching rate constants approximately double those of the C_40_ carotenoids. While shorter chain ones show a lower quenching, e.g., lutein, with 10 double bonds, quenching at a rate half that of β-carotene (or zeaxanthin, both with 11 double bonds) and septapreno-β-carotene (9 double bonds) having a quenching rate constant around one tenth that of β-carotene in benzene [36]. Lycopene is a more efficient quencher than β-carotene (and all other C_40_ carotenoids) in organic solvents and the reason for this has been suggested to be structural (although the difference is less marked in benzene compared a mixed solvent system containing ethanol, chloroform and water) [36,37] (see Table 1, below). There is a loss of planarity in β-carotene and other C_40_ carotenoids and xanthophylls with terminal six membered rings. This creates twisting of the rings due to steric hindrance, leading to an effective reduction in the conjugated chain length, which is not present in lycopene [38]. 

The functional groups of the xanthophylls do show a limited effect on the quenching activity, with a recent paper [40] showing those xanthophylls containing two carbonyl groups (astaxantin, capsorubin and its diacetate) to have the best scavenging ability in acetonitrile, while those containing one carbonyl group (capsanthin and its diacetate) are better able to scavenge than those with none at all. However, this is the opposite of previous research on three asymmetric xanthophylls [41,42] where adonirubin, with two carbonyl groups, showed a lower quenching rate constant for ^1^O_2_ in both benzene and deuterated methanol compared with adonixanthin and asteroidenone (both containing only one carbonyl group). This apparent disparity suggests further studies are needed.

Studies using organic solvents have also looked at the quenching abilities of *cis* carotenoids and, in benzene, they quench ^1^O_2_ less efficiently than the all-trans isomer with the rate constants decreasing for β-carotene as the *cis* bond moves away from the center of the molecule, e.g., from 13.5 × 10^9^ dm^3^ mol^−1^ s^−1^ for the all-trans isomer to 8.99 × 10^9^ dm^3^ mol^−1^ s^−1^ for the 9-*cis* isomer [36]. A time-resolved resonance Raman study has indicated that all β-carotene isomers share a common triplet state, twisted about the central carbon–carbon double bond compared with the ground state [43]. A recent study in hexane has shown that *cis* isomers are produced after reaction of the all-*trans* isomers with ^1^O_2_ for both lycopene and β-carotene and that lycopene can prevent β-carotene isomerization [44]. 

Studies have also been undertaken using primarily aqueous media, such as micelles [6,36] and liposomes [39,45,46] where the quenching rate constants are still found to be high (>10^8^ dm^3^ mol^−1^ s^−1^). In the liposome studies the quenching ability was independent of the site of generation (i.e., whether the ^1^O_2_ was generated by a water or lipid soluble photosensitizer). There was a marked difference in quenching ability for the xanthopylls in liposomes compared to micelles, where the rate constants fell by up to 50 times (for lutein) in liposomes, whereas the quenching rate constant for β-carotene in liposomes was close to that in micellar solution. A significant concentration effect on ^1^O_2_ quenching was also observed for xanthophylls in the liposomes (especially for zeaxanthin), suggesting aggregation lowers quenching efficiency. It is also interesting to note that, unlike in organic solvents, the difference between the quenching abilities of lycopene and β-carotene is virtually non-existent in mixed micelles of Triton-X 100 and Triton-X 405 and in dipalmitoyl phosphatidylcholine (DPPC) liposomes (see Table 1, above). Note that lycopene is virtually insoluble in Triton-X 100 micelles alone, so was not studied in this media [6].

One of the liposome studies, in dimyristoyl phosphatidylcholine (DMPC) [46] also monitored inhibition of ^1^O_2_-induced lipid peroxidation and showed that inhibition by β-carotene, canthaxanthin and astaxanthin was similar but that the inhibition by lycopene was 10-fold less. A more recent study has also shown inhibition of ^1^O_2_-induced plasma lipid oxidation by β-carotene and fucoxanthin [47], with fucoxanthin being the better inhibitor, despite the ^1^O_2_ quenching rate constant of fucoxanthin being reported to be lower than that of β-carotene [48].

Cellular studies have shown differing results; carotenoids efficiently quench ^1^O_2_ in isolated photosystem II reaction centers [49] and can also protect ex vivo lymphocytes from ^1^O_2_-induced damage [50,51,52], though the decrease in ^1^O_2_ lifetime is more easily observed for lymphoid cells which have been incubated with carotenoids and then washed [50,51], rather than when individuals have taken the carotenoids orally over several weeks [51,52].

Additionally, a recent study [53] using microscopy to observe the time-resolved ^1^O_2_ luminescence in single HeLa cells has shown no change in the lifetime of intracellular ^1^O_2_ in the presence of β-carotene, even in D_2_O where the ^1^O_2_ lifetime is significantly lengthened (15–40 μs) compared to water (~3 μs) [54]. Thus, these workers suggest that the protective effects of β-carotene observed in their cell environment may be due to radical trapping and not direct ^1^O_2_ quenching, and they propose this is due to a very low diffusion rate within the high viscosity intra-cellular environment. The ^1^O_2_ lifetime in a single cell in water is around 3 μs, which is similar to the lifetime in pure water of 3.1–4.2 μs [55]. This and the D_2_O effect on its lifetime in a single cell also suggests a low diffusion rate, otherwise it would be quenched more effectively by endogenous quenchers such as lipids and proteins.

### 3.2. Carotenoid and Xanthophyll Radicals

Free radicals, of course, are characterized by an unpaired electron. When a free radical interacts with a carotenoid several possible modes of reaction arise and these depend mainly on the nature of the free radical. The situation is, therefore, much more complex than the quenching of ^1^O_2_ by carotenoids.

Many of the studies of the generation and reactivities of carotenoid radical cations, and anions, up to 2015 have been reported in several reviews [56,57,58]. 

One of the most well studied species is the carotenoid radical cation obtained via abstraction of an electron from the carotenoid by an oxidizing free radical.

CAR + R^•^ → CAR^•+^ + R^−^

Typical examples of the strongly oxidizing free radicals that lead to this electron transfer process are: chlorinated peroxyl radicals, such as CCl_3_O_2_^•^, nitrogen dioxide (NO_2_^•^ arylperoxyl radicals, sulfonyl radicals (RSO_2_^•^) and dibromine radical anion (Br_2_^•−^). However, as we will discuss below, the strongly oxidizing hydroxyl radical (OH^•^) mainly adds to the carotenoid rather than producing the carotenoid radical cation. Furthermore, CCl_3_O_2_^•^, and possibly NO_2_^•^, may give both the radical cation and also add to the carotenoid.

Radical anions (CAR^•−^) can be generated from sufficiently reducing radicals, for example, via addition of the solvated electron (e^−^) to the carotenoid (CAR):

CAR + e^−^ → CAR^•−^

The properties and reactivity of several carotenoid radical anions, including the reactivity of carbonyl containing carotenoid anions with water, have been reviewed previously [42] and some others are briefly mentioned below. However, the radical anions are not thought to be as biologically important as the radical cations. Quenching of oxidizing free radicals, which can arise via normal metabolic processes or via environmental hazards, such as smoking and air pollution, is of considerable biological importance and therefore, the carotenoid radical cations have been studied much more extensively than the carotenoid radical anions.

The radical cations (CAR^•+^) can be generated in various ways, such as via pulse radiolysis [59,60,61], flash photolysis [62,63,64], and electrochemically [65,66]. Pulse radiolysis is also a convenient method to generate and characterize the radical anions. These techniques are well established and have allowed the one-electron oxidation potentials of several carotenoids to be measured in aqueous micellar solution—they are typically near 1000 mV, so that carotenoid radical cations, are rather strong oxidizing agents [60,61] themselves. This data shows that carotenoid radical cations may oxidize important bio-substrates such as cysteine, tyrosine and tryptophan and also allowed electron transfer between one carotenoid and the radical cation of another to be observed for many pairs of carotenoids. Therefore, we were able obtain the relative one-electron oxidation potentials of several important carotenoids showing that lycopene has the lowest potential (i.e., is the most easily oxidized of the dietary carotenoids). This may lead to lycopene being the “sacrificial” carotenoid in vivo, when there is a mixture of carotenoids present, and may well be related to the health benefits often claimed for dietary lycopene as we have previously discussed [56].

Another important result, discussed in previous reviews, for example [58], arises from pulse radiolysis studies showing that carotenoid radical cations are converted back to the parent carotenoid by water-soluble antioxidants such as ascorbic acid. Therefore, a potentially damaging pro-oxidant effect due to the high reduction potential of carotenoid radical cations will be removed by ascorbic acid. Smokers have low levels of ascorbic acid, and free radicals from cigarette smoke can reach the lungs. It has been shown that, for heavy smokers, a high concentration of β-carotene can have a damaging effect, and a speculation is that this may be due to these smoke-based free radicals (e.g., NO_2_^•^) reacting with β-carotene to generate the β-carotene radical cation, which can then damage biomolecules. 

Recent results from Skibsted and co-workers [63,64] are consistent with the above reactions of β-carotene radical cation with tyrosine and tryptophan, regenerating the parent β-carotene. Interestingly, Skibsted used pH conditions where the redox potentials (the standard reduction potentials) were the same for tyrosine and tryptophan (the reduction potential for β-carotene radical cation is independent of pH in the region studied). These workers found that tyrosine reacted an order of magnitude faster than tryptophan and speculate that this may account for tyrosine, rather than tryptophan, as the protein moiety reacting with β-carotene in the protective mechanism, which operates in the photosynthetic reaction center. As Skibsted points out, the driving force in these reactions depends on the “local” pH and in proteins the reverse reaction between a tyrosine radical and β-carotene may also be important.

In their most recent work [64] Skibsted and co-workers have extended their studies of the regeneration of β-carotene from its radical cation to eugenol and isoeugenol—naturally occurring phenols that may well be important in the stability of carotenoids in various food products. The redox potentials are 0.75 V and 0.66 V (vs. SHE) for eugenol and isoeugenol respectively however the corresponding rate constants for the “repair” of β-carotene are 4.3 × 10^9^ dm^3^ mol^−1^ s^−1^ and 7.2 × 10^8^ dm^3^ mol^−1^ s^−1^. Therefore, even though isoeugenol is the most reducing it reacts faster with the βCAR^•+^. Skibsted explains this result in terms of the so-called “inverted region” of Marcus theory. 

A recent review [67] has highlighted a significant apparent disagreement concerning the fate of β-carotene radical cation. El-Agamey and co-workers [68] studied the effect of pH on the decay of the β-carotene radical cation (HCAR^•+^) while the extensive work of Kispert and co-workers used advanced electron paramagnetic resonance techniques and optical measurements beside electrochemical and theoretical studies [67,69,70,71]. The Kispert group showed that proton loss from β-carotene radical cation leads to the neutral carotenoid radical (CAR^•^)

HCAR^•+^ → CAR^•^ + H^+^

with an absorption maximum around 750 nm. In their studies, Kispert et al. form the radicals on silicate-based matrices [67,69,70,71]. Proton loss from the radical cation to produce a neutral species was identified using both electron paramagnetic resonance and optical detection. It should be noted that this work established the optical absorption spectrum for the neutral species without using transient absorption spectroscopy. This assignment of the neutral species in the absence of oxygen was confirmed via studies of the effect of pH and this species was linked to a previously unassigned peak reported from spectral studies of Photosystem II (PSII) [69]. The Kispert group speculate that in PS II itself photoprotection can arise both by the loss of the excess vibrational energy of the radical cation and by quenching of excited chlorophyll by the carotenoid proton loss neutral radical. However, El-Agamey [68] used laser flash photolysis to generate the β-carotene radical cation and study its transient absorption in aqueous Triton-X micelles. They observed the decay of the radical cation as a function of pH and conclude that such neutral radicals of β-carotene show no absorption at wavelengths above 550 nm. 

The work of El-Agamey concerns β-carotene in Triton X100 “solutions” while that of Kispert concerns a very different microenvironment with the carotene radicals stabilized on silica-alumina or molecular sieves. Therefore, for example, diffusional processes may be more important in the solution studies of El-Agamey, while they will be unimportant in the organized microenvironments. While this may account for the different results and conclusions, clearly more work is needed to understand these apparently contradictory, but important, observations.

El-Agamey and McGarvey have extended their research on the microenvironment of carotenoids using pulsed laser studies of micro-emulsions [72]. These preparations allow the water/cyclohexane ratio to be changed, so that the polarity of the microenvironment of the carotenoid can be varied, and this in turn was found to affect the ratio of the carotenoid radical cation to other, probably neutral radicals, formed. The authors used 266 nm pulsed laser excitation of air-saturated solutions to generate the peroxyl radicals from both water soluble 4-acety-4-phenylpiperidene hydrochloride and lipid soluble 1,1-diphenylacetone. They studied two carotenoids, *zeta*-carotene and 7,7’dihydro-β-carotene and both led to the formation of the corresponding radical cation and another, unidentified, species absorbing also in the near infra-red but at somewhat shorter wavelengths (possible an ion pair or radical cation isomer) called NIR1 with wavelengths in the 650–750 nm region. Two important findings were (i) the nature of the peroxyl precursor used (water soluble or lipid soluble) had little or no influence on the yields and kinetics of the transient species formed from the reaction of the two carotenoids with the different peroxyl radicals and (ii) the ratio of the radical cation formed to NIR1 varied significantly with the environment polarity, this suggesting that the micro-emulsion composition will have a great impact on the pro-oxidant/anti-oxidant activity of carotenoids.

We have previously reviewed the generation, and reactivity of carotenoid radical anions [42]. As mentioned, these are of less relevance to biological processes than the radical cations. There are many differences between the anions and cations. For example, β-carotene radical anions react with oxygen at near diffusion-controlled rates (for several isomers in hexane) presumably to generate the superoxide radical anion, while there is no reaction between β-carotene radical cations and oxygen:

CAR^•−^ + O_2_ → CAR + O_2_^•−^

Interestingly, the corresponding reaction of oxygen with lycopene radical anion is 10 times slower (1 × 10^8^ dm^3^ mol^−1^ s^−1^) whereas there is little difference between the rates of ^1^O_2_ quenching by lycopene and β-carotene as discussed earlier.

It is also worth noting the difference in protonation/deprotonation between the carotenoid radical cations and anions. The radical anions of carbonyl containing xanthophylls (HCAR=O^•−^) react with water and methanol to generate a neutral radical in which the carbonyl oxygen is protonated [73].

HCAR=O^•^^−^ + H_2_O → HCAR-OH^•^ + OH^−^

Of course, this is quite a different species to the neutral radical generated by deprotonation of a carotenoid radical cation, discussed above:

HCAR^•+^ → CAR^•^ + H^+^

Therefore, as also discussed above, neutral carotenoid radicals can arise from deprotonation of the corresponding radical cation or protonation of the corresponding radical anion. However, another important route to neutral carotenoid radicals is via radical addition to a carotenoid.

A well-studied series of carotenoid radical adducts concerns carotenoids reacting with sulfur containing radicals, thiyl (RS^•^) and thiyl sulfonyl (RSO_2_^•^), e.g.,

CAR + RS^•^ → RS–CAR^•^

Much of this early work has come from the groups of Willson (e.g., [74]) and Skibsted and Mortensen (e.g., [75]) and has been reviewed previously [41].

An extension of this work comes from the studies of El-Agamey and McGarvey who have observed [20] the first direct reversible oxygen addition to a carotenoid-derived carbon-centered neutral radical. These workers used phenylthiyl radicals (PhS^•^) to add to 7,7’dihydro-β-carotene (and to β-carotene). The corresponding adduct PhS-77DH^•^ being sufficiently long-lived (no endo- or epoxidation processes occurring) to allow the rate of reversible oxygen addition to be observed for the first time. Typical addition rate constants are in the region of 10^4^ dm^3^ mol^−1^ s^−1^ but are about 7 times slower for β-carotene compared to 7,7’dihydro-β-carotene (presumably related to the different conjugated chain lengths).

There has been recent interest in the reactions (trapping) of the important radical OH^•^ with carotenoids. Nishino and co-workers have used electro-spray ionization, time-of-flight mass spectrometry and Electron Spin Resonance (ESR) spectroscopy to study the products of several carotenoids reacting with OH^•^ [33,40]. In all cases carotenoid epoxides were formed with a single oxygen atom adding across the 5,6 double bond of the carotenoid ring rather than on to the polyene chain. By contrast ^1^O_2_ added as two oxygen atoms forming various endoperoxides.

As noted above the strongly oxidizing hydroxy radical (OH^•^), reduction potential 2.31 V vs. SHE (Standard Hydrogen Electrode) at pH 7 [76], mainly adds to carotenoids to give neutral radical adducts rather than undergoing electron transfer to produce carotenoid radical cations. Additionally, hydrogen atom abstraction by OH^•^, also yielding a carotenoid neutral radical, has been observed [77].

It is also well established that, once formed, neutral radicals can add molecular oxygen forming peroxyl radicals and, as first shown by Burton and Ingold [78] this can lead to a switch from anti-oxidant to pro-oxidant behavior of carotenoids.

A similar mechanism was suggested by Boehm and co-workers to explain their recent observation of a substantial effect of oxygen concentration on the protection of human cells against γ-radiation by lycopene [79]. In this study, human volunteers either took a high lycopene diet or near zero lycopene and the extracted blood lymphoid cells were exposed to high energy γ-radiation from a ^60^Co source. Cell membrane destruction, leading to immediate cell death, was measured via cell staining with eosin. Under normal atmospheric conditions and at the radiation doses studied (up to 5000 Gy) the lycopene protected the cells by a factor of 4–5 compared to the unprotected cells (no lycopene in the diet). However, a really dramatic effect of oxygen concentration was observed. At near zero oxygen there was virtually total cell protection by the lycopene (no cell damage due to the high energy γ-radiation) whereas at 100% oxygen the lycopene gave no protection whatsoever. It was suggested that the molecular mechanism for this oxygen effect was related to the observations of Burton and Ingold [78] in non–biological conditions. Therefore, the proposed mechanisms involved, for example, cell protection via scavenging of the reactive OH^•^ by lycopene (either by addition or hydrogen abstraction) followed by oxygen addition to give a reactive peroxyl neutral radical. The reactive peroxyl radical then causes the cell membrane destruction. 

Whatever the precise molecular mechanism (e.g., involving one or more neutral lycopene radicals followed by oxygen addition) this huge difference in cell protection, due to oxygen concentration, may (with appropriate clinical techniques) lead to a mitigation of damage caused by radiation treatment of tumors. From a simplistic point of view (and following a high lycopene diet), flushing the tumor with oxygen should have no effect on the radiation therapy while flushing the non-necrotic regions, with say nitrogen, may led to significant protection against the unwanted radiation damage.

This oxygen addition reaction may also offer an alternative explanation for the damaging effect of high doses of β-carotene in heavy smokers (increase in lung cancers) discussed above. If the NO_2_^•^ (or other radicals) present in cigarette smoke can also add to β-carotene (as well as oxidize it), then the high concentration of oxygen in the lungs will increase the likelihood of oxygen addition to the neutral radical adducts, producing a reactive carotenoid peroxyl radical. However, since it seems likely that the NO_2_^•^ adduct (if formed) is short-lived, this may not be an important process [74], but, of course, it could be important for other radicals present in cigarette smoke.

Mildly oxidizing radicals, such as alkylperoxyl radicals, frequently react with carotenoids via adduct formation and/or hydrogen abstraction and generate a neutral carotenoid radical.

Hydrogen abstraction from a carotenoid to a free radical leads, of course, to the same carotenoid neutral radical as deprotonation of the corresponding radical cation

HCAR → CAR^•^ + H^•^

HCAR^•+^ → CAR^•^ + H^+^

and, as noted above there is interest in such neutral radicals because of their possible formation in PSII [67,69,70,71]. 

The detection of neutral radicals following hydrogen atom abstraction is not easy, but, as mentioned above, has been reported by Chen et al. [77] for OH^•^ reacting with β-carotene. This identification was based on an extremely weak and short-lived transient detected at 750 nm with a lifetime of around 150 ns—the assignment is partly based on the wavelength being in the region where neither the radical cation or radical adducts absorb.

The most recent study of hydrogen abstraction [80] concerned the extremely slow (over 10’s of hours) reaction of an aroxy radical—2,6 di-t-butyl-4-(4’-methoxyphenyl)phenoxy (AO^•^, a rather stable radical used as a model for radicals of biological interest)—with fatty acids and with 6 carotenoids. The hydrogen abstraction rate constants were reported for astaxanthin (1), β-carotene (2), lycopene (3), capsanthin (4), zeaxanthin (5) and lutein (6). These rate constants increased in the order 1 < 2 < 3 < 4 < 5 < 6 with values ranging from 8.3 × 10^−4^ dm^3^ mol^−1^ s^−1^ for lutein to 2.2 × 10^−4^ dm^3^ mol^−1^ s^−1^ for β-carotene. No value was given for the extremely slow reaction with astaxanthin. These values for the allylic hydrogen abstractions from the 6 carotenoids were explained in terms of the structures of the carotenoids and, in particular, the differing types of hydrogen atoms (based on their positions relative to π-electrons) in the parent carotenoid. It must be noted that the spectral changes reported in this work are very tiny indeed, typically from the data given, an absorption reduction of 0.025 for β-carotene after 22 h reaction time with AO^•^.

While more work on this potentially important route to neutral carotenoid radicals is worthwhile progress is hindered by the difficulties in detection of such radicals—the transient from OH^•^ reacting with β-carotene and the carotenoid spectral changes reported after reaction with AO^•^ being good examples of this problem.

## 4. Conclusions

The near infrared emission and subsequent decay of singlet oxygen is not difficult to study. Furthermore, the carotenoid/xanthophyll and retinoid radical cations are also not difficult to detect and study in appropriate solvents (the spectral properties, absorption and emission bands have virtually no experimental problems associated with spectral overlap). As a result, much is now understood of the reaction of carotenoids protective ability against photo-damage via singlet oxygen and also of the properties of carotenoid/xanthophyll and retinoid radical cations. These radical cations themselves are rather strongly oxidizing species and are able to oxidize other important bio-substrates. Regeneration of a parent C_40_ (dietary) carotenoid from the corresponding radical cation by reducing agents such as ascorbic acid has been reported. It has been suggested that detrimental effects of carotenoid radical cations (generated via environment pollutants, for example) on human health may arise when concentrations of reductants such as ascorbic acid are low. The other radicals of carotenoids, the neutral radicals formed via hydrogen abstraction processes or via radical addition, are more difficult to study for spectral and kinetic reasons. While a carotenoid radical cation does not react with oxygen, a neutral radical or a neutral radical adduct can add molecular oxygen to generate peroxyl radicals, which are likely to be damaging species.

## Figures and Tables

**Table 1:** Comparison of the ^1^O_2_ quenching rate constants for lycopene and β-carotene in a range of environments.

	*k*_q_/10^9^ dm^3^ mol^−1^ s^−1^				
Carotenoid	DPPC Liposomes [39]		Micelles [36]	Benzene [36]	Ethanol:Chloroform:Water 50:50:1 [37]
	*	†			
Lycopene	2.4	2.3	2.0	17.0	31.0
β-Carotene	2.3	2.5	2.4	13.0	14.0

* = Water soluble ^1^O_2_-generation by rose Bengal; † = Lipid soluble ^1^O_2_-generation by 4-(1-pyrene)butyric acid; DPPC: dipalmitoyl phosphatidylcholine.
